# Metaheuristics Algorithm-Based Optimization for High Conductivity and Hardness CuNi_2_Si_1_ Alloy

**DOI:** 10.3390/ma18051060

**Published:** 2025-02-27

**Authors:** Jarosław Konieczny, Krzysztof Labisz, Satılmış Ürgün, Halil Yiğit, Sinan Fidan, Mustafa Özgür Bora, Ş. Hakan Atapek

**Affiliations:** 1 Department of Railway Transport, Faculty of Transport and Aviation Engineering, Silesian University of Technology, 40-019 Katowice, Poland; krzysztof.labisz@polsl.pl; 2 Faculty of Aviation and Astronautics, Aviation Electrical Electronics, Kocaeli University, Kocaeli 41001, Türkiye; urgun@kocaeli.edu.tr; 3 Department of Information Systems Engineering, Kocaeli University, Kocaeli 41001, Türkiye; halilyigit@kocaeli.edu.tr; 4 Faculty of Aviation and Astronautics, Airframe and Powerplant Maintenance, Kocaeli University, Kocaeli 41001, Türkiye; sfidan@kocaeli.edu.tr (S.F.); ozgur.bora@kocaeli.edu.tr (M.Ö.B.); 5 Laboratory of High Temperature Materials, Department of Metallurgical and Materials Engineering, Faculty of Engineering, Kocaeli University, Kocaeli 4100, Türkiye; hatapek@kocaeli.edu.tr

**Keywords:** CuNi_2_Si_1_ alloy, aging, electrical conductivity, hardness, metaheuristics algorithms, optimization

## Abstract

The optimization of CuNi_2_Si_1_ alloy’s mechanical and electrical properties was achieved through a combination of experimental approaches and metaheuristic algorithms. Optimizing hardness and electrical conductivity through a variation in aging temperature (450–600 °C) and aging duration (1–420 min) was taken under consideration in the present work. Cold rolling with 50% strain after solution annealing aided in microstructure refinement and accelerated Ni_2_Si precipitates’ development, and property improvement increased. Optimum temperature and holding period were 450 °C and 30 min, respectively, with 266 HV and 13 MS/m and 167 HV and 11.2 MS/m for non-deformed samples, respectively. SPBO, genetic algorithm (GA), and particle swarm optimization (PSO) metaheuristic algorithms were considered, and SPBO exhibited the best prediction accuracy. SPBO predicted 450 °C for 61.75 min, and experimental testing exhibited 267 HV and 14 MS/m, respectively. Polynomial regressions with 0.98 and 0.96 values for R^2^ confirmed these values’ accuracy. According to this work, computational optimization proves effective in optimizing development and property tailoring for application in industries including aerospace and electrical engineering.

## 1. Introduction

The progress in materials engineering is continuous with respect to a need for imagination-capturing technologies and industrial growth, which require the development of new high-performance materials and the improvement of available materials. The emphasis has been given to operational durability and reliability and the use of materials that have specific and stable thermal, electrical, and mechanical properties. Improved technology and methodologies in research have assisted the processing of such materials. This development is best described by copper alloys, which possess improved qualities for a wide range of industrial needs [1]. Development of copper alloys widely used in electrical engineering, power engineering, electronics, and aeronautics is aimed at the achievement of superior properties—like increased hardness and abrasion resistance—while maintaining or slightly reducing electrical conductivity [2]. High-strength copper alloys find their applications in the automation industry, the automobile industry, and the electrical and electronic industries [3]. Miniaturization of electronic and electrical components and rising material costs drive the development of high-performance copper alloys for automotive, railway, electrical engineering, and ICT industries. These applications call for an alloy with excellent mechanical properties and medium to high electrical conductivity. The components should be resistant to stress, relaxation, and fatigue for reliability under extreme operating conditions and should exhibit property stability even under load over the temperature range from −40 °C to 180 °C [4]. Cu−2Ni–1Si alloys, which can be modified by heat and plastic treatments, are increasingly being studied with modifiers like Cr, Cr–Mg, or Cr–Ti. These modifications improve mechanical properties and, in some cases, conductivity, but strengthening frequently degrades electrical performance during heat or plastic treatments [5]. Knowing the course of processes run in an alloy during its solidification permits the microstructure and functional properties of copper alloys to be customized and maximized, accordingly. These realizations have the influence of shifting towards shorter temperatures and time for heat and plastic treatment. Larger cooling rates promote advantageous dispersion of phases and reduce block and dendritic segregation by improving interfacial distribution and fragmentation of eutectics. CuNi_2_Si_1_ alloy often contains modifiers like Cr, Cr–Mg, or Cr–Ti and, due to its variable mechanical properties, which are controlled by heat and plastic treatment, is the object of growing investigation [6]. Heat treatments of supersaturation at 900–1000 °C, aging at 400–600 °C, and others are generally applied in order to accomplish precipitation hardening of CuNi_2_Si copper alloy. Methods of processing joining heat treatment with cold plastic deformation refer to supersaturation followed by cold rolling and then aging. Aging above 600 °C is infrequent for periods of over five hours. However, such treatments have a dramatic impact on properties related to applications of industrial importance. Importantly, aging at 650 °C for more than five hours improves both tribological wear resistance and corrosion resistance in the open air, making these conditions worthwhile for particular property requirements [7]. CuNiSi alloys owe their popularity to a quite exceptional formability in treating techniques by metal forming—rolling, extrusion, and forging. In industry, there are two more widely used technological ways of making, such as semi-continuous casting followed by hot extrusion into rods and additional metal forming and continuous casting followed by any method of metal-forming procedures, such as die forging or rolling. Originally related to continuous or semi-continuous casting, both methods provide semi-products with specific macrostructures and a spectrum of material properties. Such diversity of manufacturing routes allows the mechanical and physical properties of the alloy to be tailored to meet specific application requirements, thus extending its applicability to a wide range of industrial applications [8]. The hot-working temperature range for CuNiSi alloys is relatively narrow: 850–875 °C. Due to their small heat capacity and high thermal conductivity, especially for the workpieces in Routine II, it is difficult in practice to precisely manage hot-working conditions. An alternative in this regard is the deformation that accompanies isothermal treatment. In both Routines I and III, temperatures lower than 800 °C or higher than 900 °C (for a short period only) and a straining degree above 0.7 are found to result in industrially undesirable adverse effects on microstructural refinement and homogeneity. A more sophisticated Routine III improves process reliability, with emphasis on controlled cold deformation with precisely chosen isothermal temperature and duration to optimize microstructure [9]. Electrical conductivity is generally decreased through doping, but the optimization process provides a more efficient way for improving the overall characteristics of this material. Finer-grained structures outperform coarse-grained structures due to extensive research on thermo-mechanical treatments and advanced techniques such as SPD. However, such elaborate techniques are impracticable on large workpieces. Less complicated methods of pre-deforming and aging in a sequence, such as cold rolling and forging, are widely used in practice. Despite the very long-standing interest in the interaction between deformation, precipitation, and properties [10], comprehensive studies of how pre-aging deformation might affect precipitation behavior and variations in properties and of treatment-aging interactions have been relatively rare until recently.

After hot and cold rolling and heat treatments at 400 °C and 500 °C, the mechanical and electrical characteristics of Cu-5.98Ni-1.43Si and Cu-5.98Ni-1.29Si-0.24Ti alloys were investigated. Due to denser δ-Ni_2_Si precipitates and decreased solubility of Ni and Si in the Cu matrix, Cu-5.98Ni-1.29Si-0.24Ti demonstrated higher electrical conductivity (57.1% IACS) and Vickers hardness (315.9 HV). Despite the existence of coarse Ni-Si phases from casting, Ti-alloying improved these characteristics by encouraging fine precipitates and creating Ni-Si-Ti particles [11]. Phase change during early aging was investigated by fabricating a Cu–6.0 Ni–1.0 Si–0.5 Al (wt%) alloy. B2 β-NiAl precipitates were produced by aging at 400 °C, whereas δ-Ni_2_Si, β-Ni_3_Si, and γ′-Ni_3_Al precipitates were produced by aging at 450 °C. Discontinuous δ-Ni_2_Si was formed at 500 °C. Atom probe analyses showed that Ni-Si clusters of 1–2 nm size were found at 450 °C with segregation of Al. Based on orientation maps developed, the alloy exhibited a tensile strength of 1080.2 ± 20.4 MPa, and its IACS conductivity was 25.4% after one hour at 450 °C [12]. The hardness, electrical conductivity, and microstructure of a Cu–2.9%Ni–0.6%Si alloy were studied using HPT and aging. HPT (~150 nm) resulted in a significant improvement in Vickers hardness and polished grains. Both hardness and conductivity increased with an increase in aging to 40% IACS at 100 h. Atom probe and high-resolution transmission electron microscopy demonstrated that strengthening resulted from grain refinement and nanoscale Cu_3_Ni_5_Si_2_ and NiSi precipitates. The alloy’s improved mechanical and electrical performance after HPT processing was facilitated by these precipitates [13]. The mechanical characteristics and microstructural development of the Cu–8.0Ni–1.8Si–0.15Mg alloy aged at 450 °C were examined. In early stages, aging consisted of processes such as spinodal decomposition and precipitation of β-Ni_3_Si. Long-term treatments resulted in precipitations of β-Ni_3_Si and δ-Ni_2_Si after 30 min. The alloy was characterized by a shallow dimple and a quasi-cleavage fracture pattern. After solution treatment at 970 °C for 6 h and subsequent aging at 450 °C for 180 min, the following mechanical properties were given: 1005 MPa tensile strength, 768 MPa proof strength, 5.6% elongation, and 31.5% IACS conductivity [14]. The microstructure and properties of Cu–Ni–Al alloys were studied with the addition of Sn and Si solutes using CuxNi_3_Al, Cux(Ni_2.75_Si_0.25_)Al, and Cux(Ni_2.75_Sn_0.25_)Al alloys (x = 12, 16, 24). Si was fully dissolved into γ/γ′ phases, enhancing hardness and conductivity through dual-nanostructure strengthening and improvement in thermal stability. Sn partially dissolved, reducing thermal stability but aiding γ′ phase precipitation. Si outperformed Sn in promoting thermal stability, making it more suitable for multi-component Cu–Ni–Al alloy designs [15]. With an emphasis on precipitate forms and crystal relationships, the discontinuous precipitation behavior of Cu-10Ni-1Si alloy aged at 500 °C and 550 °C was examined. Continuous δ-Ni_2_Si precipitates were formed by initial aging, followed by simultaneous development of discontinuous β-Ni_3_Si and continuous δ-Ni_2_Si precipitates. After two-hour aging at 550 °C following 87.5% cold rolling, the ultimate tensile strength of the alloy attained 1364 MPa, with a yield strength of 1295 MPa and IACS conductivity of 38.7%. In contrast to continuous-phase alloys, where Tan δ values stayed constant, discontinuous precipitates improved damping performance, showing the unique thermal response of the discontinuous phase in Cu-Ni-Si alloys. Tan δ values increased with temperature [16]. Cr, Fe, Mo, and Zr were minor alloyed into Cu-Ni-Si alloys to increase hardness without sacrificing conductivity. The optimal composition formula [(Ni,Si,M)-Cu_12_]Cu_3_ was created using a cluster-plus-glue-atom model, guaranteeing precise δ-Ni_2_Si precipitation. Alloys Cu_93.75_(Ni/Zr)_3.75_Si_2.08_(Cr/Fe/Mo)_0.42_ had ≥35% IACS conductivity and >1.7 GPa hardness. HV = 2.5–2.7 GPa and conductivity of 35–36% IACS were demonstrated by Cu_93.75_Ni_3.54_Si_2.08_(Cr/Fe)_0.42_Zr_0.21_, which is equivalent to high-strength KLFA85 alloy and confirms effective minor-alloying for enhanced performance [17]. High strength and electrical conductivity were determined afterward in Cu-Ni-Si alloys, and a Cu-6.0Ni-1.0Si-0.5Al-0.15Mg-0.1Cr alloy was thoroughly studied [18]. The alloy attained 1097.5 MPa tensile strength and 26.4% IACS conductivity following a sequence of treatments that included homogenization, hot rolling, solution treatment, cold rolling, and annealing. Multi-phase precipitation, including δ-Ni_2_Si, β-Ni_3_Si, β-NiAl, and γ′-Ni_3_Al, was discovered by transmission electron microscopy. Orowan, solution, Hall–Petch, and work hardening mechanisms were used to increase strength. A route to sophisticated, high-performance Cu alloys is provided by the multi-phase precipitation and trace alloying elements technique.

The acceleration of material discovery with machine learning has been an area of growing recent activity. It consists of constructing mathematical models describing the quantitative relationships between the features and properties of materials, based on either experimental or computational data. These models can predict all possible materials with diverse combinations of features, which would allow for the screening of candidates that meet design constraints and possess desired qualities. These materials can further be fabricated and tested in a laboratory. The ANN-GA optimization strategy has been widely employed in engineering processes since these two techniques can deal with nonlinear relationships among the influencing parameters. The ANN technique learns from practical data and trains itself by adjusting weights to estimate the optimum conditions. In contrast, GA is a population-based evolutionary search and optimization process. For instance, in most cases, the output that comes from the use of ANN is in turn used as input while applying GA, and combining outputs of the two techniques normally leads to better optimization results [19]. The principles of GA combined with ML accelerated the design using copper alloys with high electrical conductivities and strengths. The study applied the machine learning model of Catboost to establish the relationship between alloy composition, from binary to multi-component, and target attributes with an accuracy as high as 93.5%. Pareto front ideas and data augmentation were used to develop optimized compositions. In addition, advanced machine learning modeling predicted the composition with desired qualities, confirmed by independent literature data. It also showed very good agreement between experiments and predictions in providing a reliable method of predicting changes in conductivity, microstructure evolution, and mechanical properties and thus holds promise for effective alloy creation using machine learning [20]. Kolev [21] proposes a hybrid model of deep learning and ensemble learning to predict the effect of chemical composition and thermomechanical processing on characteristics in Cu–Ni–Si alloys. The model, based on the integration of the most relevant input parameters, predicts the mechanical characteristics and electrical conductivity with almost perfect R2 values. Compared to the three other machine learning models, the performance of the DL-EL model is much better. The feature significance analysis identified the most critical features driving alloy behavior. This work optimizes alloy design and processing by using predictive machine learning, therefore filling key knowledge gaps in Cu–Ni–Si alloy development and improving materials science. The traditional methods of trial and error for creating high-performance alloys are extremely time-consuming. Cu-Ni-Co-Si alloys are intriguing prospects for next-generation integrated circuits. Pan et al. [22] accelerated alloy development with a machine learning-based material design methodology. A composition-process-property database was established for Cu-Ni-Co-Si alloys, and the best candidates were screened using a multi-performance screening approach. The Cu-2.3Ni-0.7Co-0.7Si alloy outperformed the C70350 alloy by retarding the aging process and enhancing phase dissolution, hence improving stability and extending applicability. Traditional material design methods rely on human expert intuition or try different sets of compositions or parameters over and over until a required property is produced, which is often high-cost and labor-intensive. Wang et al. propose a high-throughput ML approach toward the development of models using compositional design and property predictions [23]. Desired tensile strengths of 600–950 MPa and 50.0% IACS electrical conductivity can be achieved with copper alloys designed accordingly using the system. The good agreement between the predictions and the literature data and newly synthesized alloys has demonstrated the power of ML-driven property-oriented design in such complicated high-performance materials. Qin et al. have proposed a multi-objective design approach using machine learning to enhance both ultimate tensile strength and electrical conductivity for Cu-Ni-Si alloys [2]. The methodology consists of five steps: dataset creation, feature generation, key feature screening, modeling and inverse design, and experimental iteration. It implies that a bounded dataset allows for the alignment of the compositions and properties, especially when Co is added. From a joint expectation improvement function, the alloy design improved over the initial Pareto frontier after five ML-experiment iterations. Microstructural analysis confirmed that the optimal alloy indeed exhibited superior precipitation strengthening. The development of a high-performance alloy is not easy because the exploration spaces of composition and process parameters are huge. Li et al. introduce a machine-learning-driven alloy design approach based on feature engineering and Bayesian optimization for Cu-Ni-Si alloys [24]. Characteristic extraction, error screening, and EI calculation are involved in the three-step design approach, which might serve effectively in the screening of alloy systems for synergistic strength, conductivity, and ductility. Combining process parameters will quantify the “composition-process-properties” link and enable the identification of the best Cu-Ni-Co-Si-Mg and Cu-Ni-Co-Si-Zn alloys. It provides a scalable approach for the optimization of numerous material systems with features in conflict and accelerates the design of alloys when only limited datasets are available. The strength–electrical conductivity trade-off is important in designing high-performance copper alloys. Traditional methods are normally time-consuming and can hardly be efficient, but fortunately, ML has already started offering a different perspective. Based on 407 copper alloy datasets, Zhao et al. introduced an enhanced ML design system (MLDS) [25]. This work enhanced the many-to-many predictions to a many-to-one framework using error backpropagation neural networks, tree models, and support vector machines. MLDS integrates particle swarm optimization, yielding coefficients of determination as high as 0.98 for property prediction and 0.87 for composition prediction, hence assuring excellent predictive performance and stability. The method significantly advances the design of alloy composition not only for copper but also for other materials. In this paper, the investigation of the CuNi_2_Si_1_ alloy for the optimization of hardness and electrical conductivity was carried out using advanced metaheuristic algorithms: genetic algorithms, particle swarm optimization, and artificial bee colony methods. In contrast to previous studies, experimental data are integrated in this study with comprehensive computational optimization of the influence of aging temperatures and durations. This helps with the contribution to the literature on an optimization framework that can add weight to enhancing material properties by better industrial decisions in such industrial domains as electrical engineering and manufacturing relying on high-performance copper alloys.

This study presents a novel direction by integrating metaheuristic algorithms (GA, PSO, and SPBO) and experimental thermal-mechanical processing in a bid to optimize CuNi_2_Si_1_ alloy’s mechanical and electrical performance. Unlike most studies, which have employed computational simulation and experimental testing in a mainly one-way approach, in this research, both methods have been effectively integrated, and a more effective and efficient optimization process resulted. Furthermore, successful application of SPBO to predict material performance is another innovative direction of this research, proposing an efficient and low-cost model for application in aerospace and electrical engineering fields.

## 2. Experimental Procedure

### 2.1. Material, Applied Thermo-Mechanical Treatments, and Property Determination

In order to describe the mechanism and kinetics of precipitation in the CuNiSi alloyed copper, tests were performed on samples prepared in two heat treatment variants (I) and alternating heat treatment and cold plastic deformation (II). These tests were used to perform a preliminary analysis of the mechanism of decomposition of the supersaturated solid solution in CuNi_2_Si alloyed copper. The results of the Vickers hardness tests (HV) and the results of electrical conductivity tests © [MS/m] using the Foerster Sigmatest (Foerster Instruments, Incorporated, Pittsburgh, PA, USA) were used to assess the effectiveness of the supersaturation and aging processes. The research was carried out on the commercial CuNi_2_Si_1_ alloyed copper which the chemical composition shown in Table 1.

Samples of CuNi_2_Si alloyed copper for laboratory tests were prepared in two variants.

First variant (I):

- Hot rolling with an 80% degree of deformations, up to a thickness of 3.0 mm;
- Solution annealing: heating at 950 °C for one hour and cooling in water;
- Aging at 450 °C, 500 °C, 550 °C, and 600 °C for 1 min, 5 min, 10 min, 15 min, 30 min, 60 min, 120 min, and 420 min.

Second variant (II):

- Hot rolling with an 80% degree of deformations, up to a thickness of 3.0 mm;
- Solution annealing: heating at 950 °C for one hour and cooling in water;
- Cold rolling with a 50% degree of deformations;
- Aging at 450 °C, 500 °C, 550 °C, and 600 °C for 1 min, 15 min, 30 min, 60 min, 120 min, and 420 min.

Diffraction investigations and thin foil structure investigations were performed on a transmission electron microscope JEM 3010UHR from the JEOL company (JEOL Ltd., Tokyo, Japan) with an accelerating voltage of 300 kV. Micro and nanostructure studies of thin films were performed using a high-resolution transmission electron microscope (TEM) on Titan 80/300 from FEI (FEI Company, Hillsboro, OR, USA) using the bright and dark field techniques and SAD diffraction at an accelerating voltage of 300 kV equipped with the Oxford EDS LINK ISIS X-ray energy dispersive spectrometer (Oxford Instruments NanoAnalysis, High Wycombe, UK). Thin films were made from strips of a cut piece of the sample, which was then thinned. In the next step, discs with an outer diameter of 3.2 mm were cut from the strips, which were finally thinned using an ion polisher.

Hardness measurement was performed using a Vickers hardness tester with a diamond indenter on the HV0.5 hardness scale. Measurements were performed according to the ASTM E92 standard [26]. Hardness values were averaged from 10 measurements for each sample.

The electrical conductivity of alloyed copper samples was measured according to the IEC 60028 standard [27]. Electrical conductivity values were averaged from 10 measurements for each sample. The problem of electrical conductivity measurement errors was solved by introducing a mechanism for compensating the coil’s distance from the surface of the tested element, the so-called F. Förster mechanism [28].

The Foerster Sigmatest 2.070, which was used to measure electrical conductivity, was powered by a current of 3000 mA at a voltage of 5V DC. The meter can measure in the frequency range of 60/120/240/480/960 kHz. The measurement range was 0.5 to 65 MS/m or 1 to 112% IACS, and the resolution was ±0.1% of the measured value, with automatic distance compensation of up to 750 µm. The Forster Sigmatest 2.070 m used for the measurement was characterized by a tolerance of 0.5% of the measured value, which was taken into account during the analysis of the obtained results. The Foerster Sigmaster was calibrated by the manufacturer and recalibrated during routine periodic inspection.

The Vickers hardness tester was also calibrated during routine periodic inspection. The measurement was performed ten times for each sample, and the results were averaged. The standard deviation of the average measurements oscillated in the range of 5–7%; however, due to the number of measurements of both electrical conductivity and current measurements, they were not included.

### 2.2. Mathematical Modeling

Curve fitting CF is a method generally utilized for the construction of a mathematical model from experimental data and thus provides an effective way to analyze the complex relationship between variables. Using different curve fitting methods, the derived curves ought to satisfy various constraints regarding smoothness, accuracy criteria, and physical requirements [29]. Therein, in this manuscript, MATLAB (R2021a) Curve Fitting (CF) has been employed for the handling of curve fitting because the complexity of the dataset must be dealt with in different respects. The polynomial fitting method was chosen as the best method to obtain an optimum curve fitting through preliminary testing. The MATLAB CF tool is capable of nonlinear regression in three-dimensional space: two input parameters and one output parameter can be modeled as paired relationships. These were combined using polynomial functions to produce a comprehensive model. The polynomial order for approximation was set to 3 for the *x*-axis and 4 for the *y*-axis in order to get the best approximation that reflects the experimental data with a minimum error. The selection of third and fourth order polynomials was done according to an iterative optimization process using the MATLAB Curve Fitting Toolbox. Different polynomial orders were tried and tuned until the best statistical performance metrics were obtained. Comparison of model accuracy was done on the basis of key statistical indicators, i.e., R^2^, adjusted R^2^, RMSE, and SSE values. Higher order polynomials were attempted to capture the nonlinear interaction among aging temperature, time, hardness, and electrical conductivity. However, in order to prevent overfitting and ensure generalizability, the order of the polynomial was restricted to the lowest order that produced the best fit. Accordingly, the polynomial regression Equations (1)–(4) are presented as developed based on the experimental data listed in Table 2.

To serve as a check on the statistical reliability of the experimental data in Table 2, additional statistical analysis was conducted on undeformed and deformed samples. In the undeformed and aged samples (N=24N=24N=24), electrical conductivity had a mean of 10.57 MS/m, a standard deviation of 1.06, and a 95% confidence interval (CI) of (10.12, 11.01) MS/m. The mean and standard deviation of the hardness were 147.58 HV5 and 40.21 HV5, respectively, with a 95% confidence interval of (130.60, 164.55) HV5. In the deformed and aged samples (N=20N=20N=20), electrical conductivity had a mean of 13.08 MS/m, a standard deviation of 2.47, and a 95% CI of (11.92, 14.23) MS/m. Similarly, the mean and standard deviation of the hardness were 226.6 HV5 and 31.94 HV5, respectively, with a 95% CI of (211.65, 241.55) HV5. The data indicate that deformed samples have greater electrical conductivity and hardness than undeformed ones, supporting the strengthening effect of plastic deformation. The confidence intervals also assure the statistical validity of the data, ensuring that the reported values are representative and reproducible.

f_hardness = 3122 − 19.62 x − 90.75 y + 0.041 x^2^ + 0.573 xy − 0.2214 y^2^ − 2.801 × 10^−5^ x^3^ − 0.001052 x^2^y − 0.0008351 xy^2^ + 0.006574 y^3^ + 6.509 × 10^−7^ x^3^y + 3.17610^−7^ x^2^y^2^ + 3.188 × 10^−6^ xy^3^ − 3.686 × 10^5^ y^4^ (1)

This is a polynomial function of two variables, x (temperature) and y (time), containing coefficients for each term representing the hardness, f_hardness. The terms will involve linear, quadratic, cubic, and quartic interactions between x and y. Coefficients determine the value of each term that goes to make up the value of f_hardness.

f_con = 148.7 − 0.8753 x − 1.096 y + 0.0018 x^2^ + 0.00728 xy − 0.00653 y^2^ − 1.22 × 10^−6^ x^3^ − 1.173 × 10^−5^ x^2^y − 2.35 × 10^−6^ xy^2^ + 0.0001707 y^3^ + 5.435 × 10^−9^ x^3^y + 3.192 × 10^−8^ x^2^y^2^ − 5.234 × 10^−8^ xy^3^ − 6.427 × 10^−7^ y^4^ (2)

Given here is the equation of a polynomial with two variables, x and y, to represent conductivity f_con. It also has linear and different orders of interactions, such as quadratic, cubic, and quartic, of the variables x and y, similar to the case in the hardness function, where coefficients are representative of respective contributions to the overall conductivity by each term.

f_def_hardness = −1762 + 10.8 x + 42.81 y − 0.01936 x^2^ − 0.1961 xy − 0.17 y^2^ + 1.147 × 10^−5^ x^3^ + 0.0003556 x^2^y − 0.0001414 xy^2^ + 0.00247 y^3^ − 2.428 × 10^−7^ x^3^y + 4.137 × 10^−7^ x^2^y^2^ − 1.121 × 10^−6^ xy^3^ − 8.388 × 10^−6^ y^4^ (3)

It defines the two variables, x and y, in the form of the following polynomial function to represent the f_def_hardness involving its linear and quadratic, cubic, and quartic interactions in terms of the coefficient values representing their respective contributions to deformation hardness.

f_def_con = 153 − 0.8496 x − 3.624 y + 0.001643 x^2^ + 0.02031 xy − 0.001222 y^2^ − 1.051 × 10^−6^ x^3^ − 3.246 × 10^−5^ x^2^y − 5.842 × 10^−5^ xy^2^ + 0.0002459 y^3^ + 1.785 × 10^−8^ x^3^y + 3.009 × 10^−5^ x^2^y^2^ + 1.487 × 10^−7^ xy^3^ − 1.416 × 10^−5^ y^4^ (4)

The above is the deformation conductivity, f_def_con, as a function of two variables, x and y, represented as a polynomial that includes linear terms, quadratic, cubic, and quartic terms, and coefficients that determine how each interaction contributes to the deformation conductivity.

One of the most important criteria for the suitability of a regression model is how close the predicted values are to the observed data. This criterion can be measured by several metrics. In this study, RMSE, SSE, R-squared, and adjusted R-squared values, as calculated using the MATLAB Curve Fitting tool, were used for this purpose. The R^2^ values reflect the fitting accuracy of this model, hence showing how much reality there is between the real data and the predicted outcomes. An R^2^ close to 1 means that a relationship has been almost perfectly fitted, and this therefore means that the values of the model have had a close relationship with the observed data [30,31,32].

The calculated SSE for hardness was 294 HV5, and that for conductivity came out to be 1.2. Since hardness ranges between 60–267 HV5 and conductivity goes from 8.6–15.2 MS/m, this small number of values of SSE makes quite a great fit in the case of model fitting corresponding to each dataset in consideration. Accordingly, for the hardness and conductivity characteristics of the samples, there were two R-squared (R^2^) values: 0.98 and 0.96, respectively. Very good agreement was indeed accomplished between the experimental results and the polynomial fitting by obtaining rather high adjusted R-squared values of 0.95 and 0.96 for hardness and conductivity, respectively, to ascertain reliability in the fit developed for this polynomial model. In addition, the respective RMSE values were 7 and 0.4 for hardness and conductivity, respectively. These low RMSE values are further confirmation of how well the curve fitting was performed, which indicates that the selected polynomial approach is a proficient way to align the developmental trends hidden in the data.

A thorough comparison between experimental and predicted values has been conducted in an attempt to assess the accuracy of optimized values. Polynomial regression models utilized in work generated high values for correlation, including R^2^ = 0.98 and adjusted R^2^ = 0.96 for prediction of hardness and R^2^ = 0.96 and adjusted R^2^ = 0.95 for prediction of electrical conductivity. In addition, RMSE values for prediction of hardness and electrical conductivity were 7 and 0.4, respectively, confirming a strong model-data fit. Low values for SSE (294 for hardness and 1.2 for conductivity) confirm the proposed scheme for optimization. All these values confirm that the metaheuristic model for optimization accurately forecasts the property of a material with negligible deviation in experimental values, confirming high accuracy and robustness in performance.

## 3. Implementation and Results

### 3.1. Microstructural Impacts of Cold Rolling and Aging

**Figure 1:**
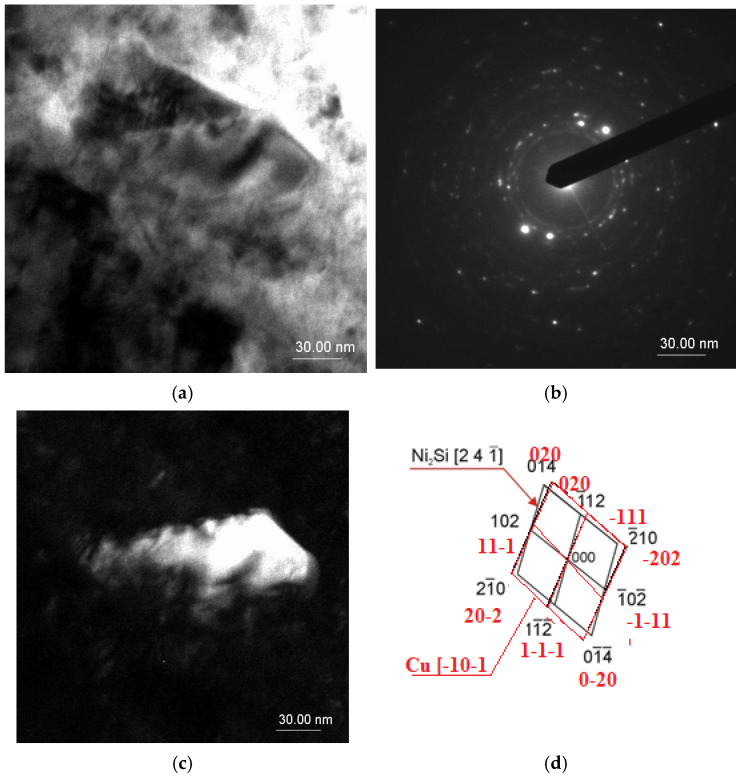
Structure of the CuNi_2_Si_1_ alloy: (**a**) bright field image; (**b**) diffraction pattern from the area as in Figure (**a**); (**d**) solution of the diffraction pattern on Figure (**b**); (**c**) dark field image forming the $24\underline{1}$ spot of the Ni_2_Si phase (P6322 spatial group) recrystallizing in a hexagonal lattice.

The use of cold rolling with 50% work hardening after solution annealing had a significant impact on the alloy structure. Since the precipitation of secondary particles occurs in privileged places, which include grain boundaries or crystal lattice defects (vacancies, dislocations), cold plastic deformation caused an increase in the number of secondary particle nucleation sites [33]. In the case of the CuNi_2_Si alloy, the secondary particles responsible for alloy strengthening are the Ni_2_Si phase [5]. In the CuNi_2_Si alloy, which was not plastically deformed to cold but only heat treated (solution annealing and aging), there are fewer privileged places for the precipitation of the secondary phase; therefore, after the precipitation of the Ni_2_Si phase during aging at 600 °C, a very rapid growth of the precipitated particles occurred after 10 min (Figure 1). The zone axis of the determined Ni_2_Si phase is set as [2 4 −1] with a quite optimal quality factor of 0.35. Based on the electron diffraction pattern, it can be stated that the phase is not coherent with the Cu matrix, because of a lack of planes on the diffraction pattern that are parallel–coherent—or even semi-coherent to the determined intermetallic phase [22]. This crystallographic relationship causes an additional strengthening factor of enhancement of the mechanical properties based on the creation of internal stress of the first type—between very small areas ranging around the interplanar distance of the crystallographic cells (Figure 1d).

**Figure 2:**
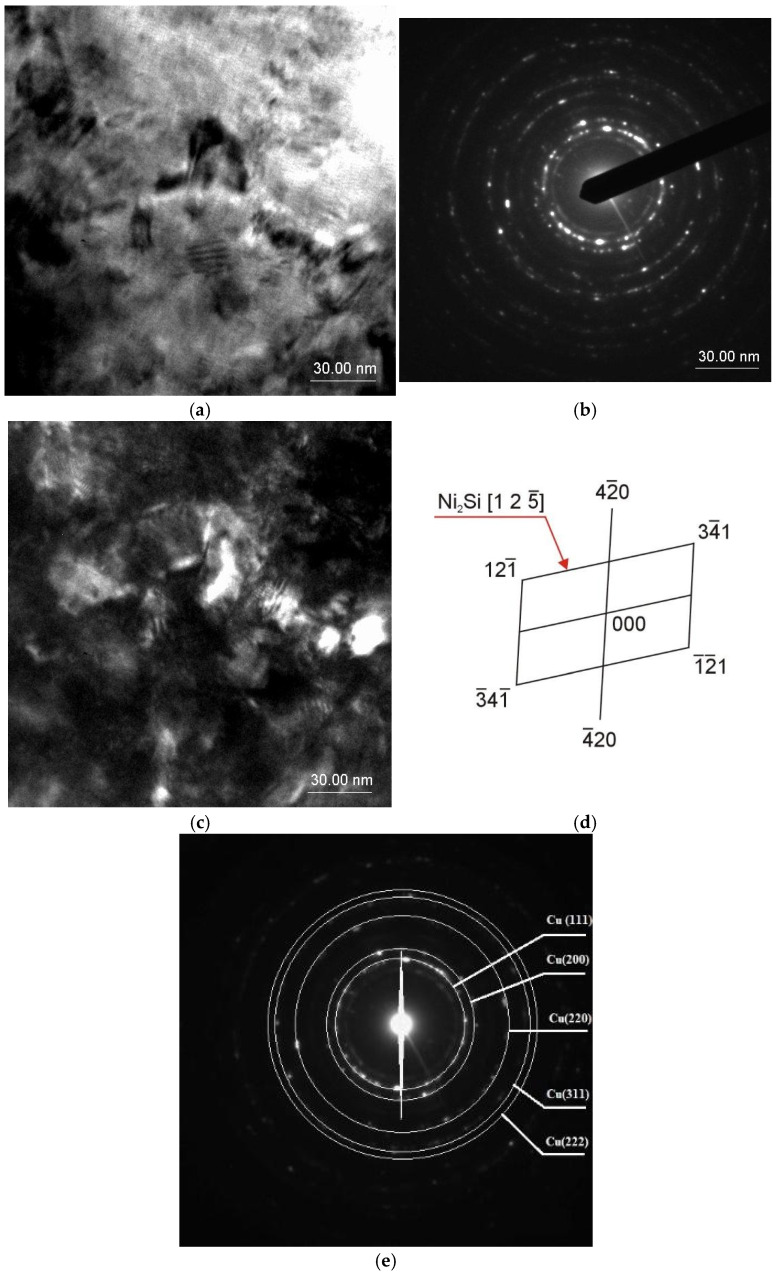
Structure of the CuNi_2_Si_1_ alloy: (**a**) bright field image; (**b**) diffraction pattern from the area as in Figure (**a**); (**d**,**e**) solution of the diffraction pattern on Figure (**b**); (**c**) dark field image forming the $12\underline{5}$ spot of the Ni_2_Si phase (P6322 spatial group) recrystallizing in a hexagonal lattice.

The zone axis of the determined Ni_2_Si phase is set as [2 4 −1] with a quite optimal quality factor of 0.35. Based on the electron diffraction pattern, it can be stated that the phase is not coherent with the Cu matrix, because of a lack of planes on the diffraction pattern that are parallel–coherent—or even semi-coherent to the determined intermetallic phase. This crystallographic relationship causes an additional strengthening factor of enhancement of the mechanical properties based on creation of internal stress of the first type—between very small areas ranging around the interplanar distance of the crystallographic cells (Figure 2d).

In the CuNi_2_Si alloy, as a result of cold rolling (Z = 50% reduction) after solution heat treatment, a large number of structure defects were formed, and the share of grain boundaries in the structure increased, which are privileged places of Ni_2_Si phase nucleation. For this reason, a larger number of grains are separated during aging, which are smaller (Figure 2) than in the case of classical heat treatment.

In the CuNi_2_Si alloy, because of cold rolling (Z = 50% reduction) after solution heat treatment, a large number of structure defects were formed, and the share of grain boundaries in the structure increased, which are privileged places of Ni_2_Si phase nucleation [13]. For this reason, a larger number of grains are separated during aging, which are smaller (Figure 2) than in the case of classical heat treatment. The precipitation process during aging after cold plastic deformation after solution heat treatment (solution heat treatment-cold rolling-aging) proceeds in a completely different way than during aging after solution heat treatment [34]. This is related to the simultaneous precipitation (nucleation and grain growth) of the second phase, in this case Ni_2_Si, and also to the thermally induced recovery process. In this case, the minimum energy necessary for nucleation of the second phase during aging is much lower than in the case of solution heat treatment and aging [35]. For these reasons, the precipitation of the second phase and, as a result, modeling of the final properties such as hardness and electrical conductivity of the tested alloy are more difficult.

Crystal structure defects, such as vacancies or internodal atoms, as well as, e.g., dislocation clustering or the Orovan mechanism result in an increase in stacking fault energy, which on a macro scale translates into an increase in hardness and conductivity, while reducing plastic properties [36,37,38]. Similar research results for the CuTi4 alloy are presented in [39] on pages 67–73 and 75–80.

Enhancing mechanical properties through the creation of internal stress of the first type (also known as residual stress) is a well-documented phenomenon. For example, some key pieces of evidence supporting this claim are as follows:

- Stress-Induced Phase Transformation: In materials like medium-carbon low-alloy steel, internal stresses can trigger phase transformations, such as the transformation-induced plasticity (TRIP) effect. This effect enhances both strength and plasticity by transforming retained austenite into martensite under stress.
- Injection-Molded Products: Internal stresses formed during the cooling process of injection-molded products significantly influence their mechanical properties. These stresses can lead to improved strength and dimensional stability by creating a complex stress distribution within the material.
- Thermodynamic Analysis: Studies using thermodynamic methods and molecular dynamics simulations have shown that internal stresses can drive phase transitions in materials, contributing to enhanced mechanical properties. For example, the stress-induced martensitic transformation in steels is a result of internal stress [40].

### 3.2. Optimization Performance of Metaheuristic Algorithms

The processes of optimization are hugely dependent on proper definition and formulation of the fitness function, which is a basic constituent in metaheuristic optimization algorithms. A fitness function acts like a guide to move the search toward optimal solutions. However, due to some inherent defects or complexities within the problem itself, an ideal solution may not be reached. These challenges indicate that the design of the fitness function has to be done with due care to obtain robust and reliable results. In this work, the fitness function is mathematically defined in Equation (5) for the optimization of laser process parameters. This provides a structured approach to effectively evaluate and refine the process parameters. This function not only addresses some practical limitations of the process of optimization but also allows a substantial improvement in the algorithm convergence towards a viable solution.

fitness = |f_(hardness_des) − f_hardness| + |f_(conductuvity_des) − f_conductuvity| (5)

Here, f_harness_des and f_conductuvit_des, are the desired output parameters to be obtained. f hardness and f conductivity are the polynomials in Equations (1)–(4).

This study employed four metaheuristic algorithms: genetic algorithm (GA), gray wolf optimization (GWO), particle swarm optimization (PSO), student psychology-based optimization (SPBO), teaching–learning-based optimization (TLBO), and whale optimization algorithm (WOA). Table 3 presents the hyperparameters utilized for each optimization method.

The selection of metaheuristic algorithm hyperparameters was guided by values in relevant documented studies considered best for similar categories of optimization problems. Adopted values in Table 3 included values such that efficient convergence and the dangers of early stagnation and high computational expense could be avoided. For instance, a population value of 100 was adopted, having been proven in relevant studies to represent an effective value in balancing diversity in solutions and computational efficiency. In a similar manner, PSO’s cognitive (c1) and social (c2) coefficients at 2, in harmony with values long established in practice, allowing for an even balancing between exploration and exploitation, were adopted. Inertia weight was lowered linearly from 0.9 to 0.2 in an effort to enhance convergence dynamics and mitigate the danger of getting trapped in a local optimum. In addition, each algorithm’s hyperparameters were tuned through pilot runs in a manner that confirmed that adopted values delivered strong performance in optimizing CuNi_2_Si_1_’s hardness and conductivity. Doing this ensures that adopted hyperparameters have both theoretical and empirical performance justification.

**Figure 3:**
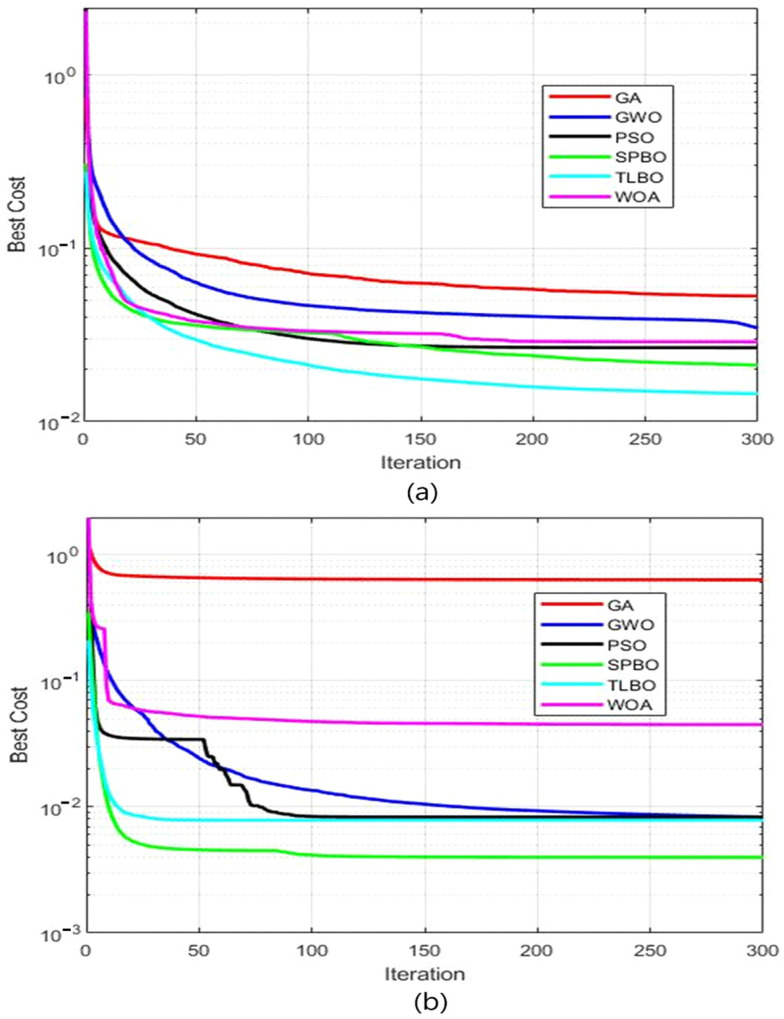
Comparison of the best cost values of different metaheuristics by iterations. (**a**) Undeformed process and (**b**) deformed process.

Figure 3 presents the comparison of the optimization performances of different metaheuristics in terms of the “best cost” metric. In general, the performance of meta-heuristics depends on the type of problem and the structure of the problem. Therefore, the choice of the most appropriate method for a given problem is crucial. It is important to compare the performances of different methods in order to choose the algorithm that best meets the requirements of the problem. The graph in Figure 3 provides useful information for such a comparison. Performance comparisons of the optimization algorithms in a non-deformed process are shown in Figure 3. When the graph is analyzed, it is seen that the student psychology-based optimization (SPBO) algorithm reaches the lowest cost value the fastest compared to the other methods. Teaching–learning-based optimization (TLBO) and particle swarm optimization (PSO) algorithms initially performed well, but their efficiency decreased after a certain number of iterations. The rest of the approaches, such as genetic algorithm, gray wolf optimization, and whale optimization algorithm, have fallen into higher costs with low optimization successes. On the other hand, for the analysis of a deformed process presented in Figure 3b, it is observed that the SPBO algorithm reaches the lowest cost value in the fastest way. However, from this graph, it was observed that the PSO algorithm had a fast start and outperformed TLBO up to some iterations. Once more, GA, GWO, and WOA algorithms have remained at higher costs and have had low success related to the optimization process. The performance of the metaheuristic algorithms may differ with respect to problem type, and process structure can also be seen with these results. SPBO exhibits a very similar performance for both undeformed and deformed processes, while other algorithms may exhibit different performances based on the structure of the process.

Table 4 shows the optimal aging temperature and times that have been identified by meta-heuristic algorithms to achieve targeted electrical conductivity, MS/m, and hardness, HV5, values for both un-deformed and deformed processes. The optimization of electrical conductivity and hardness was made using fitness functions, Equation (5), where each algorithm operated independently for the UD and D processes and recorded optimal results in the table. The information mentioned in the process section refers to the polynomial equations of the process used by the algorithms to reach the target values. Meta-heuristic algorithms produced specific solutions for un-deformed and deformed processes depending on the problem type.

For UD processes, lower values of temperature and time were generally suggested, which indicates that the optimization of un-deformed materials can be performed faster. On the other hand, the temperature and time values recommended for D processes are generally higher, indicating that deformation complicates the optimization process. However, it is noteworthy that the SPBO algorithm exhibits superior performance in both processes and offers low-cost solutions. It also follows from the comparison with other algorithms that SPBO generally reaches the optimum faster and can be more flexible. For example, GWO and WOA methods required higher temperature and time elapse to reach the values considered for target, which reduced the cost-effectiveness in some cases. Finally, the comparison of the performance of different meta-heuristic algorithms is given in Table 4, which is helpful in choosing the most suitable method for the problem at hand. The SPBO algorithm turned out to perform more successfully and stably on a wide range of problems, and it is stated that the algorithm provides a reliable solution in different processes.

When the values in the table are analyzed, it can be seen that the metaheuristic algorithms derive different optimum temperature and time values to find the desired electrical conductivity and hardness values. This shows that very different temperature and time combinations within the data space can provide the same electrical conductivity and hardness values. This diversity reveals the flexibility of meta-heuristic algorithms in the problem-solving process and their capacity to explore solution paths in the data space. In particular, it is understood that some algorithms follow different routes to achieve similar goals for different processes, so it is critical to choose the most appropriate algorithm for the problem type. This flexibility makes it possible not only to achieve target values, but also to optimize different costs and time requirements. In this context, Table 4 generally highlights how metaheuristic algorithms can follow different solution paths and find alternative optima in the data space to achieve the desired outputs. For example, the SPBO algorithm was found to have explored a larger data space for both un-deformed and deformed processes, while the solution paths of algorithms like GWO and WOA were much smaller and generally required high temperature and time. These again pinpoint the selection of an appropriate algorithm with respect to the process type and the requirement of the problem.

Table 6 shows the error percentages for electrical conductivity and hardness values obtained after using the optimum temperature and time parameters suggested by the meta-heuristic algorithms in both polynomial models and artificial neural network (ANN) models. This table provides an opportunity to analyze the performance of the algorithms in detail.

The optimization of EC and hardness factors was performed with a single-layered FFNN model in an artificial neural network (ANN) environment. The hyperparameters for training a single-layered FFNN model are discussed in Table 5. Implementation of the model involved use of the MATLAB Neural Network Toolbox software tool. To make a robust neural network model, model training was performed 1000 times in an independent manner, with each run having a randomized dataset with 80% for training, 10% for validation, and 10% for testing. In fact, with determination of EC and hardness values at an optimized level through an ensemble, EC and hardness values at an optimized level have been calculated through an average of 1000 such runs in an independent manner.

The input parameters for the artificial neural network (ANN) model involve significant processing parameters in materials science, including processing type (which is grouped into deformed and non-deformed types), aging temperature, and aging duration. All such parameters have a strong bearing on the property of the material and have been selected to effectively represent the processing condition material performance relation in a comprehensive manner. Output parameters for the ANN model involve electrical conductivity (EC) and hardness, and these are significant factors for describing the mechanical and electrical behavior of the material. Experimental values for measurement, addressed in Section 2, have been used for training the ANN, and such values have been arranged in a manner that will yield a balanced and representative distribution of input and output relations.

The model’s performance exhibited high sensitivity towards both regularization parameters and the hidden layer’s number of neurons, both of them tuned using Bayesian optimization. Bayesian optimization successfully charted hyperparameter space through iteratively selecting the most useful combination based on an acquisition function. As a result, 11 hidden layer neurons and a regularization value of 0.1 were considered to be best. To enable generalization and prevent overfitting, early stopping was implemented. That is, training was interrupted when performance in terms of validation no longer increased for 10 successive epochs, effectively preventing overtraining that could lead to model overfitting.

Table 6 serves to contrast the performances of the various metaheuristic algorithms in the prediction of optimal process parameters. The first seven rows in Table 6 are copied directly from Table 2, constituting experimentally derived values. These data points were purposely included within the validation set in order to ascertain the validity of the chosen fitness function and the formulated nonlinear regression model. The fact that these values are included guarantees that the optimization strategy accurately recreates experimental observations. The rest of the rows in Table 6 represent intermediate values that do not belong to the set of experimental data but were included to illustrate the capability of the metaheuristic algorithms in approximating any target value within the search space. In this way, their performance in getting to best values for untried data points is guaranteed. Table 6 thus proves and validates the accuracy and effectiveness of the optimization process in proving that metaheuristic algorithms can actually search through search spaces and arrive at values that closely represent desired values.

The optimum parameters found by the meta-heuristic algorithms were used in the polynomials obtained based on the experimental data, and the predictive power of these polynomials was evaluated. According to Table 6, the error rates of the values estimated by polynomials were generally at low levels. However, a wider error distribution was observed in the predictions obtained with ANN. This reflects the effect of error sources arising during the training process used in ANN models. Small deviations in the ANN’s learning of the data space were found to increase the overall error rate. Nevertheless, the lack of large differences between the polynomials and the ANN models shows the reliability of the optimum parameters found by the meta-heuristic algorithms. Table 6 also reveals the performance differences between the meta-heuristic algorithms. For example, it is noteworthy that the SPBO algorithm provides the lowest error rates in both polynomial and ANN models and exhibits superior performance in both electrical conductivity and hardness targets. Among the other algorithms, TLBO and PSO produce relatively successful results, while GWO and WOA have higher error rates in some cases. However, it is considered that the results of all algorithms are at reasonable error levels, and these differences are due to the solution search paths of the algorithms in the problem space. In conclusion, Table 6 shows that the parameters suggested by the meta-heuristic algorithms give reliable results when evaluated with both experimental polynomials and ANN models and that the algorithms are an effective tool in the optimization of processes. However, it is understood that a significant part of the errors is due to the regression operations performed during the generation of polynomials. In addition, small deviations in the learning process of the ANN also contribute to the total error rate. Nevertheless, the solutions provided by the meta-heuristic algorithms show that they offer an effective optimization strategy, maintaining acceptable levels of accuracy in both experimental and model-based analyses.

## 4. Conclusions

In this work, the mechanical and electrical properties of CuNi_2_Si_1_ were systematically optimized by metaheuristic algorithms combined with thermo-mechanical treatments. Computational analyses and experimental results showed that the combination of cold rolling with the aging process can lead to a significant improvement in microstructural characteristics and thus enhanced overall material performance. These findings present a robust framework for the optimization of critical properties like hardness and electrical conductivity that can be readily adapted for industrial applications. A significant refinement of microstructure was achieved, particularly in solution heat-treated and 50% cold-rolled samples, where Ni_2_Si precipitates preferentially nucleated at grain boundaries and lattice defects.

This refinement aided in post-subsequent aging improvement in material property. For instance, aging at 450 °C for 30 min yielded a successful improvement in conductance and in mechanical hardness, confirming processing parameters’ strong relation with performance in a material. In samples subjected to deformation and then aging under equivalent conditions, conductance and hardness showed even greater improvement, confirming mechanical deformation’s role in microstructure improvement in an alloy.

Metaheuristic algorithms, including GA, PSO, and SPBO, accurately predicted the most ideal aging parameters, successfully resolving complex interdependence between temperature, time, and mechanical deformation. SPBO operated with high efficiency, with minimum computational expense and high predictive accuracy. SPBO precisely predicted aging at 450 °C for 61.75 min to yield a 267 HV and 14 MS/m in samples subjected to deformation—results that closely approximated experimental values. Polynomial models in the environment of optimization corroborated prediction values with high values for R-squared, 0.98 and 0.96, for confidence.

This research revealed valuable insights into microstructure evolution and algorithmic optimization. Dynamic precipitation of Ni_2_Si during aging was found to be a key factor in the control of the final property of the alloy. The algorithms successfully predicted this behavior and established optimized aging conditions for a balance between strengthening through precipitation and retaining high electrical conductance. With optimized aging, a fine-grain microstructure, in which Ni_2_Si precipitates were dispersed, provided a balanced combination of mechanical integrity and electrical performance.

The findings of this work have direct engineering consequences in industries including aerospace, electrical engineering, and high-tech production. By synergistically combining experimental methodologies with computational approaches, metaheuristic approaches build a strong platform for predicting the best processing conditions with considerable savings in experimental cost and time. In the present work, an experimental and computational integration platform is presented with a demonstration of its utility in accelerated development of future-generation materials with engineered property profiles.

The innovative aspect of this work is in blending powerful computational metaheuristics with meticulous experimental testing, a practice not only for attaining predictive accuracy but also for economizing both cost and time in traditional trial-and-error development of materials. Most specifically, its application of the SPBO algorithm reflects a new level of effectiveness in simulating complex behavior in materials with little computational expense. By leveraging such computational-experimental synergy, this work introduces a new model for future work in high-performance copper alloys, with potential for accelerated and optimized development of materials with desired property profiles for commercial application.

For future development, the scheme of optimization can be extended with a broader range of alloying additions and new processes of deformation. Engaging with advanced machine learning algorithms can enable even higher predictive accuracy, allowing for real-time decisions in engineering practice. Besides, in situ observation during processes of aging and deformation can enable even deeper microstructure-evolution insights, allowing for even more accurate protocols of optimization. By bridging experimental and computational approaches, this work opens a path towards rapid development and optimization of high-performance Cu alloys for new engineering applications.

## Figures and Tables

**Table 1:** Chemical composition (wt.%) of the CuNi_2_Si_1_ alloy.

Cu	Si	Zn	P	Pb	Sn	Mn	Ni	Sb	Bi	As	Cd
97.02	0.89	0.13	0.065	0.003	0.009	0.030	2.08	0.001	0.001	0.001	0.001

**Table 2:** Experimental parameters and observed outputs.

Aging Temperature (T, °C)	Aging Time (t, min)	Condition			
		Undeformed and Aged		Deformed and Aged	
		Electrical Conductivity (MS/m)	Hardness (HV5)	Electrical Conductivity (MS/m)	Hardness (HV5)
450	1	8.6	68.7	8.6	230
	10	10.4	126	-	-
	15	10.5	132	11.8	259
	30	11.2	167	13	266
	60	11.4	180	14	267
	120	12.1	181	14.4	257
500	1	9	70	8.2	230
	10	10.7	156.5	-	-
	15	11	164.1	13.3	259
	30	11.5	185	14.3	254
	60	12.1	188	14.6	242
	120	11.8	175	15.2	212
550	1	8.7	77	8.6	230
	10	11.1	192	-	-
	15	11.4	189	14.3	248
	30	11.5	167	14.7	224
	60	11.5	157	14.9	203
	120	11.7	154	15	187
600	1	8.6	69.5	8.6	230
	10	9.6	175	-	-
	15	9.7	164	14.5	211
	30	9.8	158	14.4	197
	60	10.7	136	14.4	171
	120	10.9	129	14.7	155

**Table 3:** The hyperparameters of the metaheuristic algorithms used.

Algorithm	Parameters	Description	Value
Genetic Algorithm (GA)	Population size	The total number of individuals (solutions) in each generation	100
	Crossover rate	The probability of applying the crossover operation to selected individuals	0.8
	Mutation rate	The probability of making random changes in the genes of individuals	0.3
	Selection	The process of choosing individuals from the population for reproduction	Roulette wheel
Particle Swarm Optimization (PSO)	Swarm size	The total number of particles (candidate solution) in the swarm	100
	Cognitive coefficient (c1)	Determines the influence of a particle’s personal best on its velocity.	2
	Social coefficient (c2)	Determines the influence of the global best on a particle’s velocity.	2
	Inertia weight	Controls the influence of a particle’s previous velocity on its current one	decrease from 0.9 to 0.2
Gray Wolf Optimizer (GWO)	Population size	The total number of gray wolves (candidate solutions) in the population	100
	Alpha, beta, delta parameters	Hierarchical effect of leader wolves	Dynamic
	Random coefficients (r1 and r2)	Two random values adjusting wolves’ positions dynamically	[0, 1]
	Control parameter (a)	It balances global exploration and local exploitation	linearly decreased from 2 to 0
Whale Optimization Algorithm (WOA)	Population size	The total number of whales (candidate solutions) in the population	100
	Control parameter (a)	It balances exploration (searching globally) and exploitation (focusing locally)	linearly decreased from 2 to 0
	Random parameter (p)	A random value deciding between encircling or spiral-based position updates	[0, 1]
Teaching–Learning-Based Optimization (TLBO)	Population size	The number of learners in the population	100
	Teaching factor (TF)	The rate at which the teacher influences the learners	1 or 2
Student Psychology-Based Optimization (SPBO)	Population size	The number of students (solutions) in the population	100
	Motivation factor (M)	The student’s motivation to solve a problem, influencing their willingness to find better solutions	1.00
	Learning rate (LR)	The effect of students’ individual and group learning	0.5

**Table 4:** Optimum temperature and time parameters derived by metaheuristic algorithms to obtain the desired electrical conductivity and hardness values.

		**TLBO**			**GA**			**PSO**		
**Desired Electrical Conductivity**	**Desired Hardness**	**Process**	**Optimum Aging Temperature**	**Optimum Aging Time**	**Process**	**Optimum Aging Temperature**	**Optimum Aging Time**	**Process**	**Optimum Aging Temperature**	**Optimum Aging Time**
MS/m	HV5		ToC	t min		ToC	t min		ToC	t min
11.2	167	UD	472.879	23.88	UD	546.06	34.29	UD	472.88	23.88
11.4	189	UD	450.000	62.12	UD	542.46	20.51	UD	542.46	20.51
12.1	188	UD	512.232	63.44	UD	523.52	63.44	UD	512.24	63.44
8.6	69.5	UD	473.409	1.45	UD	480.62	1.22	UD	487.67	1.00
13	266	D	461.058	22.45	D	480.20	76.11	D	461.06	22.45
14.3	248	D	484.738	57.93	D	570.01	84.65	D	519.89	23.71
14	267	D	450.000	61.75	D	585.05	93.80	D	450.00	61.75
8.6	230	D	566.737	1.02	D	450.00	69.87	D	566.51	1.00
11	150	UD	450.611	31.75	UD	462.32	45.58	UD	557.14	41.72
12	180	UD	517.644	61.88	UD	517.90	61.55	UD	517.66	61.88
14	200	D	565.521	74.39	UD	564.41	74.19	D	564.10	47.62
13	250	D	497.217	74.54	UD	497.22	74.54	UD	497.22	74.54
		**SPBO**			**GWO**			**WOA**		
**Desired Electrical Conductivity**	**Desired Hardness**	**Process**	**Optimum Aging Temperature**	**Optimum Aging Time**	**Process**	**Optimum Aging Temperature**	**Optimum Aging Time**	**Process**	**Optimum Aging Temperature**	**Optimum Aging Time**
MS/m	HV5		ToC	t min		ToC	t min		ToC	t min
11.2	167	UD	473.05	24.85	UD	472.84	24.34	UD	472.87	23.89
11.4	189	UD	451.14	62.06	UD	542.43	20.52	UD	542.46	20.51
12.1	188	UD	512.45	63.48	UD	600.00	78.17	UD	600.00	78.17
8.6	69.5	UD	473.77	1.43	UD	473.55	1.44	UD	486.73	1.03
13	266	D	461.05	22.46	D	460.85	22.73	D	461.06	22.45
14.3	248	D	484.74	57.93	D	519.88	23.72	D	507.90	31.09
14	267	D	449.13	61.01	D	450.00	61.75	D	450.00	61.75
8.6	230	D	566.74	1.02	D	566.74	1.02	D	566.74	1.02
11	150	D	561.62	124.41	UD	450.00	31.43	UD	450.00	31.43
12	180	UD	517.79	61.90	UD	596.44	75.96	UD	597.02	76.02
14	200	D	564.07	47.65	D	564.09	47.63	D	563.88	47.90
13	250	D	521.30	12.41	D	521.29	12.41	UD	497.23	74.54

**Table 5:** The hyperparameters of the neural network used.

Parameters	Value
Training algorithm	Levenberg–Marquard
Regularization parameter	0.1
The number of hidden layers	1
The number of hidden neurons	11
Max fail (early stopping)	10
Transfer functions of hidden layer and output layer	“tansig”, “linear”
The ratio of the number of training, validation, and testing samples	0.8, 0.1, 0.1
The number of multiple runs	1000

**Table 6:** The performance of the optimum parameters proposed by the meta-heuristic algorithms in polynomial and artificial neural network (ANN) models, with error rates in electrical conductivity and hardness values.

		**TLBO Polynom**		**TLBO ANN**		**GA Polynom**		**GA ANN**		**PSO Polynom**		**PSO ANN**	
**Desired Electrical Conductivity**	**Desired Hardness**	**Optimum Elect. Cond. % Error**	**Optimum Hardness % Error**	**Optimum Elect. Cond. % Error**	**Optimum Hardness % Error**	**Optimum Elect. Cond. % Error**	**Optimum Hardness % Error**	**Optimum Elect. Cond. % Error**	**Optimum Hardness % Error**	**Optimum Elect. Cond. % Error**	**Optimum Hardness % Error**	**Optimum Elect. Cond. % Error**	**Optimum Hardness % Error**
MS/m	HV5	ToC	t min	ToC	t min	ToC	t min	ToC	t min	ToC	t min	ToC	t min
11.2	167	0.0017	0.0006	0.0391	0.0063	0.0020	0.0014	0.0368	0.0424	0.0000	0.0000	0.0399	0.0058
11.4	189	0.0013	0.0001	0.0018	0.0437	0.0099	0.0001	0.0739	0.0436	0.0099	0.0001	0.0728	0.0435
12.1	188	0.0000	0.0000	0.0547	0.0447	0.0010	0.0278	0.0611	0.0734	0.0027	0.0090	0.0569	0.0443
8.6	70	0.0122	0.0007	0.0491	0.0253	0.0124	0.0001	0.0479	0.0291	0.0131	0.0000	0.0421	0.0205
13.0	266	0.0000	0.0000	0.0626	0.0186	0.3626	0.0205	0.0977	0.0712	0.0000	0.0000	0.0622	0.0181
14.3	248	0.0000	0.0000	0.0363	0.0148	0.3098	0.1486	0.0325	0.2819	0.0037	0.0000	0.0180	0.0012
14.0	267	0.0104	0.0002	0.0306	0.0042	0.4196	0.2235	0.0521	0.3745	0.0104	0.0002	0.0304	0.0040
8.6	230	0.0001	0.0000	0.0472	0.0052	0.8327	0.1852	0.0627	0.0475	0.0014	0.0000	0.0463	0.0048
11.0	150	0.0000	0.0000	0.0072	0.1159	0.0002	0.0018	0.0164	0.1882	0.0000	0.0000	0.0165	0.1021
12.0	180	0.0000	0.0000	0.0507	0.0134	0.0019	0.0068	0.0532	0.0145	0.0000	0.0001	0.0527	0.0132
14.0	200	0.0110	0.0002	0.0012	0.0134	0.0916	0.0002	0.0470	0.0724	0.0110	0.0000	0.0021	0.0133
13.0	250	0.0000	0.0000	0.0786	0.0149	0.0000	0.0000	0.1091	0.0565	0.0000	0.0000	0.0778	0.0146
		**SPBO Polynom**		**SPBO ANN**		**GWO Polynom**		**GWO ANN**		**WOA Polynom**		**WOA ANN**	
**Desired Electrical Conductivity**	**Desired Hardness**	**Optimum Elect. Cond. % Error**	**Optimum Hardness % Error**	**Optimum Elect. Cond. % Error**	**Optimum Hardness % Error**	**Optimum Elect. Cond. % Error**	**Optimum Hardness % Error**	**Optimum Elect. Cond. % Error**	**Optimum Hardness % Error**	**Optimum Elect. Cond. % Error**	**Optimum Hardness % Error**	**Optimum Elect. Cond. % Error**	**Optimum Hardness % Error**
MS/m	HV5	ToC	t min	ToC	t min	ToC	t min	ToC	t min	ToC	t min	ToC	t min
11.2	167	0.0009	0.0025	0.0369	0.0014	0.0004	0.0014	0.0397	0.0022	0.0000	0.0000	0.0391	0.0078
11.4	189	0.0022	0.0001	0.0012	0.0436	0.0099	0.0001	0.0745	0.0443	0.0099	0.0001	0.0736	0.0435
12.1	188	0.0003	0.0003	0.0571	0.0461	0.0033	0.0001	0.0866	0.2895	0.0033	0.0001	0.0877	0.2891
8.6	70	0.0119	0.0009	0.0487	0.0289	0.0120	0.0003	0.0487	0.0324	0.0130	0.0002	0.0471	0.0242
13.0	266	0.0000	0.0000	0.0420	0.0190	0.0001	0.0003	0.0616	0.0187	0.0000	0.0000	0.0626	0.0187
14.3	248	0.0000	0.0000	0.0362	0.0146	0.0037	0.0000	0.0184	0.0010	0.0201	0.0000	0.0690	0.0086
14.0	267	0.0000	0.0000	0.0313	0.0025	0.0104	0.0002	0.0303	0.0044	0.0104	0.0002	0.0306	0.0038
8.6	230	0.0001	0.0000	0.0486	0.0047	0.0001	0.0000	0.0477	0.0047	0.0001	0.0000	0.0481	0.0037
11.0	150	0.0002	0.0002	0.0152	0.0464	0.0001	0.0000	0.0095	0.1126	0.0001	0.0000	0.0085	0.1113
12.0	180	0.0001	0.0000	0.0529	0.0153	0.0013	0.0013	0.0809	0.2507	0.0001	0.0001	0.0814	0.2513
14.0	200	0.0110	0.0000	0.0003	0.0135	0.0110	0.0000	0.0026	0.0143	0.0110	0.0000	0.0015	0.0129
13.0	250	0.0000	0.0000	0.0375	0.0158	0.0000	0.0040	0.0765	0.0154	0.0000	0.0000	0.0004	0.0151

## Data Availability

The original contributions presented in this study are included in the article. Further inquiries can be directed to the corresponding author.

## Abbreviations

The following abbreviations are used in this manuscript:

SPBO	student psychology-based optimization
IACS	International Annealed Copper Standard
HPT	high-pressure torsion
KLFA	high strength alloy- (Cu-3.2Ni-0.7Si-1.1Zn wt%, HV = 2.548 GPa)
ANN	artificial neural network
ML	multi level
MLDS	multi-level design system
DL-EL	deep learning and ensemble learning
HV	Vickers hardness tests
TEM	transmission electron microscope
SAD	selected area diffraction
EDS	energy dispersive X-ray spectrometer
FEI	Feldstein electron imaging
CF	curve fitting
GWO	gray wolf optimization
PSO	particle swarm optimization
WOA	whale optimization algorithm
TLBO	teaching–learing-based optimization
FFNN	feedforward neural network
